# Modification of Gene Expression of Tomato Plants through Foliar Flavonoid Application in Relation to Enhanced Growth

**DOI:** 10.3390/genes14122208

**Published:** 2023-12-13

**Authors:** Alberto Martinez-Alonso, Lucia Yepes-Molina, Angel L. Guarnizo, Micaela Carvajal

**Affiliations:** 1 Aquaporins Group, Plant Nutrition Department, Centro de Edafología y Biología Aplicada del Segura (CEBAS, CSIC), Campus Universitario de Espinardo, Edificio 25, 30100 Murcia, Spain; amalonso@cebas.csic.es (A.M.-A.); lyepes@cebas.csic.es (L.Y.-M.); 2 Departamento de Biología Vegetal, Facultad de Biología, Universidad de Murcia, 30100 Murcia, Spain; angelluigi.guarnizo@um.es

**Keywords:** biostimulant, phenolic compounds, cell area, hormones, RNA-seq

## Abstract

The exogenous application of phenolic compounds is increasingly recognized as a valuable strategy for promoting growth and mitigating the adverse effects of abiotic stress. However, the biostimulant effect under optimal conditions has not been thoroughly explored. In this study, we investigated the impact of foliar application of flavonoids, specifically CropBioLife (CBL), on tomato plants grown under controlled conditions. Our study focused on determining growth parameters, such as cell size, and assessing the concentration of hormones. Principal component analysis (PCA) from all physiological variables was determined. Additionally, we utilized high-throughput mRNA-sequencing technology and bioinformatic methodologies to robustly analyze the transcriptomes of tomato leaves regulated by flavonoids. The findings revealed that CBL primarily influenced cell enlargement by 60%, leading to increased growth. Furthermore, CBL-treated plants exhibited higher concentrations of the hormone zeatin, but lower concentrations of IAA (changes of 50%). Moreover, RNA-seq analysis indicated that CBL-treated plants required increased mineral transport and water uptake, as evidenced by gene expression patterns. Genes related to pathways such as fatty acid degradation, phenylpropanoid biosynthesis, and ABC transporters showed regulatory mechanisms governing internal flavonoid biosynthesis, transport, and tissue concentration, ultimately resulting in higher flavonoid concentrations in tomato leaves.

## 1. Introduction

Changing environmental conditions coupled with the growing need for sustainable agriculture to ensure future food security have intensified during the last few years. Therefore, the importance of application of biostimulants in agricultural practices is increasing. The incorporation of biobased products, including organic agriculture, employing biofertilizers, and implementing biocontrol methods, represents a significant step forward in achieving global food security sustainably [1]. Among these sustainable strategies, plant biostimulants have gained increasing attention in recent years for their potential to enhance crop productivity and resilience. These biostimulants include a diverse range of substances that, when applied to plants, promote growth and development. In this context, the tomato plant (*Solanum lycopersicum* L.) stands out as a key subject of study by the wide economic importance of the crop. Tomato is not only a globally significant crop but also a model organism for studying plant responses to various biostimulants and stressors [2].

Flavonoids, a class of secondary metabolites produced by plants, have emerged as promising candidates to act as biostimulants. Due to their relatively low molecular weight, they play integral roles in fundamental plant physiological functions and exhibit protective effects against environmental changes [3]. While previous research has primarily explored their in vitro antioxidant properties [4], recent studies have revealed their high capacity for maintaining homeostasis and regulating growth. For instance, enhanced accumulation of flavonols has been linked to the regulation of stomatal movement, facilitating concurrent photosynthesis and water uptake and transport [5]. Additionally, studies have demonstrated significant correlations between stomatal density, elevated levels of phenolic compounds and flavonoids, increased antioxidant activity, elevated chlorophyll levels, and enhanced photosynthesis [6]. Understanding the multifaceted roles of flavonoids in plant physiology, including their potential as biostimulants, is necessary. However, research on the external application of flavonoids to plants has been limited. In a recent study, we determined that foliar external application of phenolic compounds had the potential to enhance plant growth by optimizing the exchange of CO_2_ and water in tomato leaves, thus positively influencing photosynthesis and transpiration. Also, this exchange of water and CO_2_ at the cellular level was linked to the activation of aquaporins, which can induce morphological changes, including an increase in leaf stomatal density [7].

Hormonal regulation has been highlighted as a potential target for biostimulants to promote higher crop yields. For instance, the bioactive form of auxin (IAA) facilitates vegetative growth through processes such as cell expansion, cell differentiation, morphogenesis, and organogenesis. Cytokinins play a crucial role in cell division and in establishing source–sink relationships within the plant [8]. In fact, it has been reported that these phytohormones and their cross talk play a pivotal role in determining agricultural productivity [9]. Furthermore, all the aforementioned processes are highly linked to the regulation of gene expression, adding further complexity to the mode of action of the biostimulants.

Due to progress in omics sciences, significant strides have been made in recent years towards investigating the modes of action of plant biostimulants. In this sense, RNA-sequencing technology, which efficiently enables the study of global gene expression patterns in plants, stands out as the most comprehensive option for understanding the molecular mechanisms through which biostimulants influence plant growth. This technology provides deep insights into how biostimulants can modulate the expression of genes related to plant growth and development, contributing to a more complete understanding of their molecular-level functioning [10].

In this study, we investigate the effects of an externally applied purified extract of phenolic compounds as a novel biostimulant (CropBioLife, CBL, Aussan Laboratories, Campbellfield, VIC, Australia) on the growth and leaf cell size of tomato plants under hydroponic conditions. Our research focuses on elucidating how flavonoid treatment affects hormone concentrations. Additionally, we conduct an in-depth RNA-seq analysis to explore modifications in gene expression profiles resulting from CBL treatment. By addressing these key aspects, we aim to unravel the tripartite interactions among flavonoid-based biostimulants, hormonal regulation, and gene expression, providing valuable insights into their potential as tools for enhancing tomato plant growth and productivity.

## 2. Materials and Methods

### 2.1. Experimental Design and Culture Conditions

Seeds of tomato (*S. lycopersicum* L. cv Marmande from Ramiro Arnedo, Spain) were pre-hydrated with deionized water and aerated continuously for 24 h. After this, the seeds were germinated in vermiculite in the dark at 28 °C, for three days. Then, the seedlings were transferred to a controlled-environment growth chamber with a light–dark cycle of 16–8 h, a temperature of 25–20 °C and relative humidity of 60–80%. Photosynthetically active radiation (PAR) of 400 μmol m^−2^ s^−1^ was provided by LEDs (Pacific LED, WT 470C, LED8OS/840 PSD WB L1600 lights, Philips, Amsterdam, The Netherlands). Then, they were transferred to hydroponic conditions in 16 L containers (6 plants in each) filled with Hoagland’s solution, pH 5.5. The solution was continuously aerated and changed every week. The composition of the solution was: 6 KNO_3_, 4 Ca(NO_3_)_2_, 1 KH_2_PO_4_, and 1 MgSO_4_ (mM), and 25 H_3_BO_3_, 2 MnSO_4_, 2 ZnSO_4_, 0.5 CuSO_4_, 0.5 (NH_4_)_6_Mo_7_O_24_, and 20 Fe-EDDHA (μM).

After 10 days, a first foliar spray of CBL (CropBioLife, Aussan Laboratories Pty Ltd., Campbellfield, VIC, Australia, containing 12% of flavonoids) was applied (diluted to 3 mL L^−1^) to half of the plants, and after 5 days, another application of CBL was administered. Control plants were supplied with the same volume as CBL-treated ones, with distilled water. The plants remained in the growth chamber throughout the entire experiment until the sampling was conducted. The measurements and samples collection were carried out 3 days after the second foliar treatment.

### 2.2. Dry Weight

Five plants from each treatment were collected, the root from the shoot separated, and kept in an oven at 60 °C for 5 days until they were completely dry. After that, the dried plants were weighed. Finally, the shoot-to-root ratio (shoot dry weight (DW)/root DW) was calculated.

### 2.3. Light Optical Microscopy Preparations and Measurement of Cell Area

Sections from three leaves from each treatment, all of them of the same size, were cut from the middle to the base of the leaf (growth zone), including the midrib. Immediately, the sections were immersed in a fixative solution of 2% (*v*/*v*) glutaraldehyde in 0.1 M phosphate buffer (pH 7.2) under vacuum for 1 h until the sections sank and then for 12 h at 4 °C. Subsequently, the samples were dehydrated in a gradual series of acetone–water (10% steps from 30% to 90%, each for 60 min, 100% for 180 min, and finally 100% overnight). The dehydrated samples were embedded in Epon-812 resin. Semithin cross sections (500 nm) were obtained using a motorized microtome RM 2155 Leica. Finally, the sections were affixed to slides and stained with 1% toluidine blue. Micrographs were obtained using a camera (Altra 20 Olympus UCMAP3, Tokyo, Japan) attached to an Olympus CKX41 microscope. The area of cells in the collenchyma, mesophyll (palisade parenchyma and lacunar parenchyma), and epidermis was measured using the open-access image processing software Fiji/Image J (v1.53c) (https://imagej.nih.gov/ij/, accessed on 1 September 2023).

### 2.4. Hormone Extraction and Analysis

Deep-frozen ground leaf samples (0.1 g) at −80 °C were extracted from three plants for each treatment with 500 µL of a solvent solution of methanol:isopropanol:acetic acid (25:24.5:0.5) and sonicated for 30 min with vortexing every 10 min. Subsequently, the samples were centrifuged for 5 min at 16,000× *g* at 4 °C, the supernatant collected, and the pellets re-extracted with 250 µL of solvent solution. Finally, the supernatants were filtered through a 0.22 µm-diameter pore PVDF membrane. The separation and analysis of samples were performed with a high-performance liquid chromatography–mass spectrometry (HPLC-MS) system at the Service of Molecular Biology (SBM) within the Scientific and Technical Research Area (ACTI) of the University of Murcia. The analysis was performed in negative-ion mode on an Agilent 1290 Series II HPLC (Agilent Technologies, Santa Clara, CA, USA) equipped with an automated multisampler module and a high-speed binary pump and connected to an Agilent 6550 Q-TOF mass spectrometer (Agilent Technologies, Santa Clara, CA, USA) using an Agilent Jet Stream Dual electrospray (AJS-Dual ESI) interface. Experimental parameters for HPLC and Q-TOF were set in MassHunter Workstation Data Acquisition software (Agilent Technologies, Rev. B.08.00). A reverse-phase C18 column (Zorbax Eclipse Plus, 2.1 × 10 mm, 1.8 µm) was equilibrated at 25 °C. The flow rate was 0.4 mL/min and the injection volume 20 µL. The mobile phase consisted of ultrapure water MilliQ + 0.05% acetic acid (A) and acetonitrile + 0.05% acetic acid (B), starting with 1% B and using a linear gradient to obtain 1% solvent B at 1 min. The percentage of solvent B was then increased to 99% at 7 min, 99% at 9 min. Finally, the system returned to the solvent B was reduced to 1% for the last 5 min. Absorbance was recorded at 210, 254, 280, and 320 nm. Data analysis was performed with MassHunter Qualitative Analysis Navigator software (Agilent Technologies, Rev. B.08.00). The following standards were used for quantification: indole 3-acetic acid (IAA) (MedChemExpress, HY-18569, Monmouth Junction, NJ, USA), gibberellic acid (GA3) (MedChemExpress, HY-N1964), zeatin (MedChemExpress, HY-19700); and the extracted ion chromatograms were analyzed for the corresponding compounds: IAA, C_10_H_9_NO_2_, 174.0561 > 130.0650 *m*/*z*; zeatine, C_10_H_13_N_5_O, 218.1047 > 200.0946 *m*/*z*, and GA_3_, C_19_H_22_O_6_, 345.1344 > 143.0866 *m*/*z*.

### 2.5. RNA Extraction

For RNA extraction, deep-frozen samples of leaf at −80 °C were used. Each sample was 3 middle leaves mixed from 5 different plants. This process was performed using the NZY Total RNA Isolation kit (NZYtech, Lisbon, Portugal) following the manufacturer’s protocol and using 50 mg of sample. As an additional step, the ground samples were vortexed for 20 s after adding the extraction buffer. Possible traces of contaminating DNA were removed with the DNase I included in the kit. The concentration and purity of the RNA were quantified with a UV-vis NanoDrop 1000 microvolume spectrophotometer (Thermo Fisher Scientific, Waltham, MA, USA), and its integrity was verified by agarose gel electrophoresis. The extracted RNA was stored at −80 °C until analysis.

### 2.6. RNA-Seq Analysis and Differential Expression

The RNA-seq data (3 libraries from each treatment, each of them corresponding to 5 plants) previously obtained by our laboratory [7] were reanalyzed according to the following protocol. Raw read quality was assessed using FastQC software version 0.11.2 [11], and subsequently subjected to preprocessing for quality and adapter trimming using Trimmomatic version 0.39 software [12] with default parameters. Trimmed reads were then mapped to the tomato reference transcriptome “ITAG4.0,” comprising 34,076 protein-coding genes (http://solgenomics.net/, accessed on 31 September 2023), utilizing Hisat2 2.1.0 software [13]. The resulting BAM files were generated using Samtools View software version 1.9 and subsequently sorted by name with Samtools Sort software version 2.0.3. Finally, read counts were derived from alignment files using featureCounts software, a component of the Subread package 1.6.2 [14], with default parameters. This was based on the “exon” feature within the “gene_id” meta-feature of GTF annotation files obtained from Sol Genomics (http://solgenomics.net/, accessed on 31 September 2023). Reads with multiple mappings and overlapping regions were excluded from the counting process. Differential gene expression analysis between the control and CBL-treated plants was conducted using the R procedure within the Bioconductor package DESeq2 [15]. Additionally, *q*-values were adjusted using Benjamini and Hochberg’s approach to control the false-discovery rate [16]. Genes with a *p*-value (*q*-value) of ≤0.05 and an absolute value of log2 fold change (|log2FC|) of ≥2 were considered differentially expressed. Gene Ontology (GO) and Kyoto Encyclopedia of Genes and Genomes (KEGG) analysis were performed to identify the differentially expressed genes (DEGs) enriched in GO terms and metabolic pathways, respectively. To facilitate functional categorization and pathway visualization of DEGs, we utilized the GTF annotation file and Interproscan program [17] for GO term annotation and gene function. Since there is a better annotation for *Arabidopsis thaliana*, the orthologous genes with this species (Best-hit-*Arabidopsis*-name in Table S1) were used, then a transformation to Entrez Gene code was performed. The enrichment of DEGs in KEGG pathways and GO analysis was assessed using the ClusterProfile R package [18]. A corrected *p*-value (*q*-value) of ≤0.05 was set as the threshold for significantly enriched GO terms and KEGG pathways.

### 2.7. Statistical Analysis

Statistical analysis was performed using R Studio software 2022.07.2 [19]. Significant differences between the values from all parameters were determined at *p* < 0.05 according to one-way ANOVA and Student’s *t*-test. All the results are presented as the mean ± SE. R packages FactoMineR [20] and factoextra [21] were used to generate principal component analysis (PCA) results.

## 3. Results

### 3.1. Plant Growth and Cell Area

**Figure 1:**
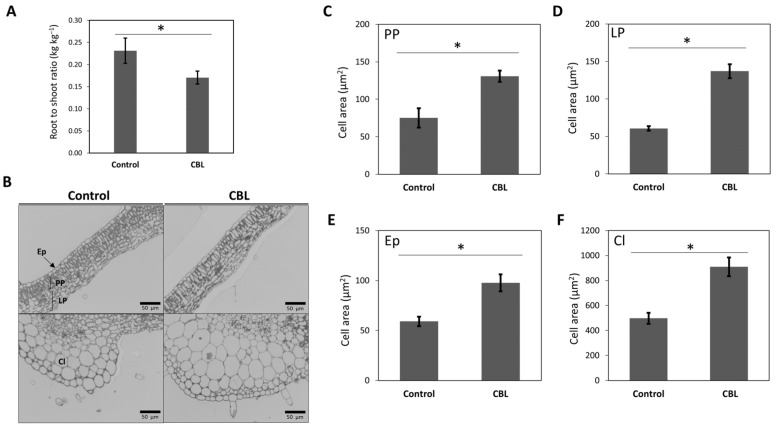
Root-to-shoot ratio (kg kg^−1^) (**A**), microscopy images of leaf cells (**B**), and cell area (µm^2^) of palisade parenchyma, PP (**C**), lacunar parenchyma, LP (**D**), epidermis, Ep (**E**), and collenchyma, **Cl** (**F**) of tomato plants under control conditions and CBL-treated. Data are means ± SE (*n* = 5 plants in (**A**), and *n* = 20–25 cells in (**C**–**F**)). Asterisks indicate significant differences according to Student’s *t*-test (*p* < 0.05).

The growth parameters of tomato plants are significantly influenced by the biostimulant CBL. Consequently, observable changes occurred in the root-to-shoot ratio (RSR) and cell area following treatment. On the one hand, RSR exhibited a statistically significant decrease, indicating vigorous growth in the aerial or productive part of the plants (Figure 1A). On the other hand, microscopic analysis of cell areas in thin sections revealed that cells in the CBL-treated plants were larger than those in the control plants (Figure 1B–F). Various cell types (palisade parenchyma, PP; lacunar parenchyma, LP; epidermis, Ep; collenchyma, Cl) were measured, all of which exhibited significant differences, with cells from leaves treated with CBL showing larger sizes in all cases, with size increases of 40% to 60%.

### 3.2. Hormone Content

**Figure 2:**
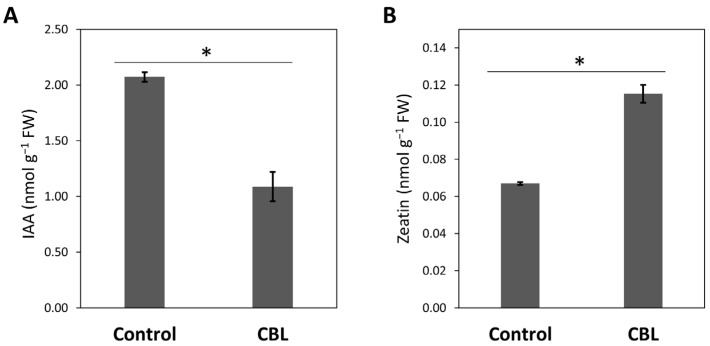
Concentration (nmol g^−1^ fresh weight, FW) of indole acetic acid (IAA) (**A**) and zeatin (**B**) in the aerial parts of tomato plants under control conditions and CBL-treated. Data are means ± SE (*n* = 3). Asterisks indicate significant differences according to Student’s *t*-test (*p* < 0.05).

Hormone content, specifically indole acetic acid (IAA) and zeatin concentration, was measured in the leaves of both control tomato plants and those subjected to CBL treatment. The findings depicted in Figure 2 show that CBL treatment brings about significant alterations in both hormones, with IAA decreasing by 48% (Figure 2A), while zeatin shows a noteworthy increase of approximately 42% (Figure 2B).

### 3.3. Global Plant Response

**Figure 3:**
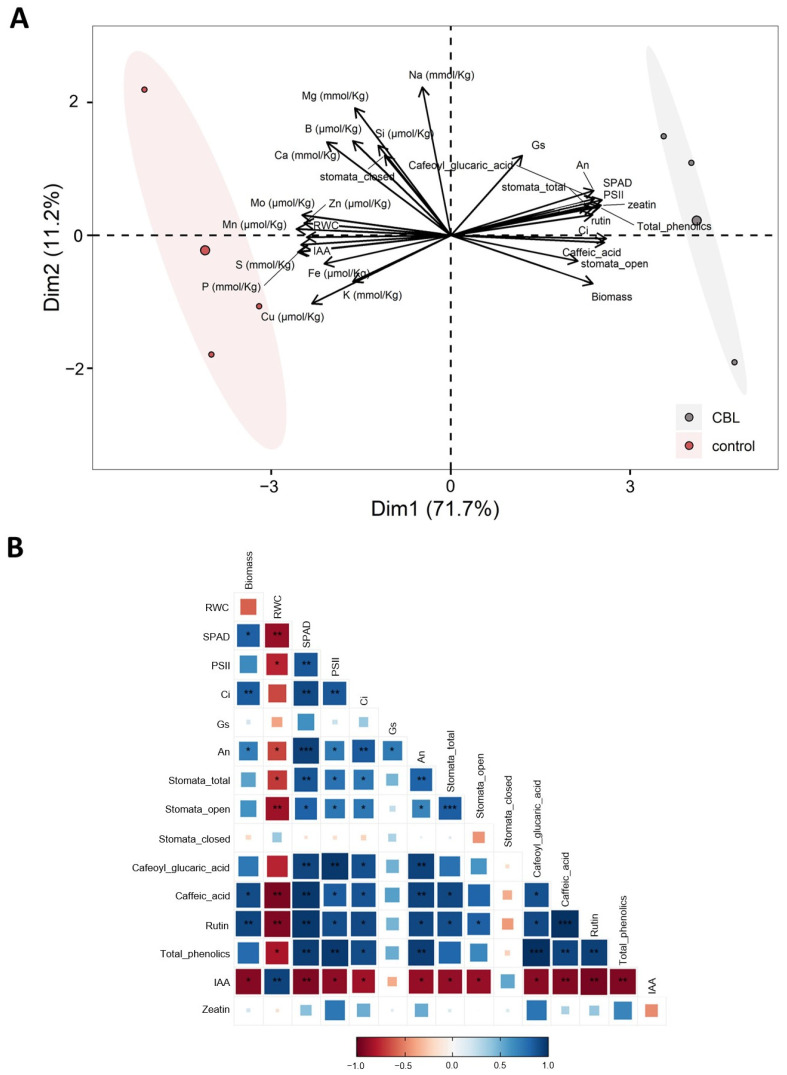
Principal component analysis (PCA) (**A**) and correlation plot (**B**) from physiological variables (micronutrients, macronutrients, gas exchange parameters, biomass and phenolic compounds from Martinez-Alonso et al. [7], and hormone content). Asterisks in (**B**) indicating: * *p* < 0.05; ** *p* < 0.01; *** *p* < 0.001.

In a study recently published [7], we determined that the exogenous application of CBL under the same conditions as in the present study had a biostimulant effect on tomato plants, as it influenced the increase in photosynthesis, CO_2_ fixation, stomatal conductance, and number of stomata. All of this led to an increase in water transport, evidenced by the upregulation of most aquaporin isoforms, and to a greater nutrient uptake by the plant. As a consequence of the CBL treatment, an increase was observed in the concentration of various phenolic compounds, such as chlorogenic acid (CGA), caffeic acid, or rutin. In order to integrate the analyses conducted in this study with those previously published [7], a PCA was performed and a correlation plot constructed (Figure 3).

From this multivariate analysis, on the one hand, a clear separation between the control group and the CBL-treated group is shown (Figure 3A). The separation is influenced by the variables that align parallel to the *x*-axis, along which the two groups are distributed, one to the right and the other to the left. Among these variables, phenolic compounds stand out, showing a positive correlation with CBL-treated plants, as is also the case with photosynthesis and biomass. It is important to note that the two hormones measured in this study, zeatin and auxin, significantly contribute to the separation of the groups. They also align in parallel to the *x*-axis, but in opposite directions. On the other hand, multiple correlations among variables are revealed (Figure 3B). Among these correlations, it is worth noting that IAA correlates negatively with almost all variables, as does the relative water content (RWC). In contrast, total phenolics show positive correlations with the rest of the variables.

### 3.4. DEG Identification

**Figure 4:**
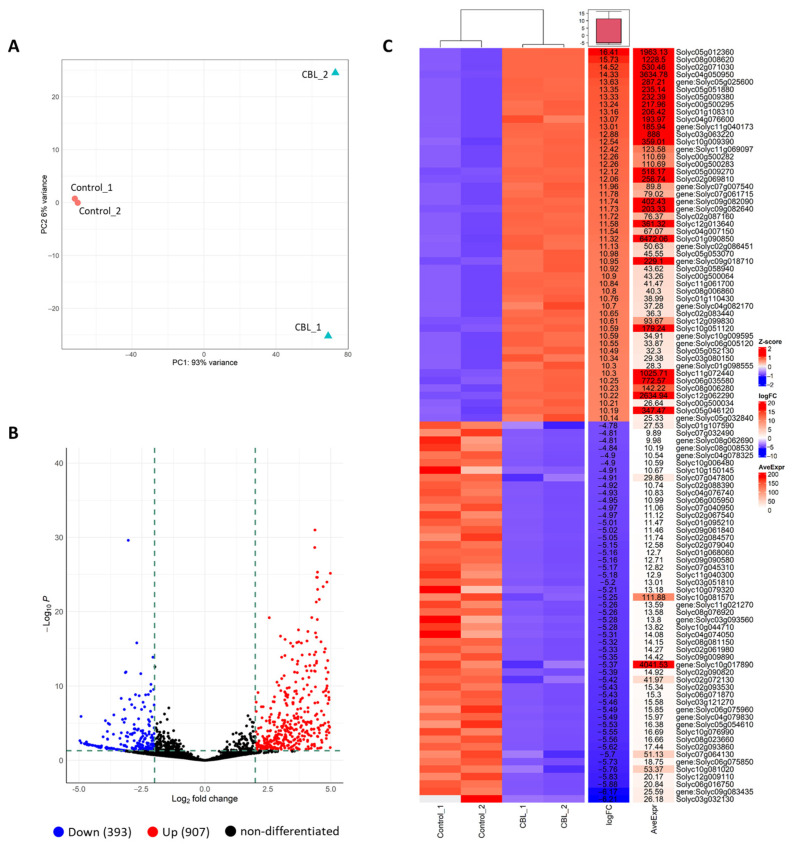
Principal component analysis (PCA) plot for RNA-seq data of biological replicates of control and CBL-treated plants (**A**). Differentially expressed genes (DEGs) are visualized using a volcano plot (**B**) for the comparison between CBL-treated and control groups. The horizontal axis represents the fold-change value (log2(B/A)) of gene expression differences between the control and CBL-treated groups, while the vertical axis represents the significance level, with *p*-values indicating the extent of gene expression changes. Each point in the plot represents an individual gene, with blue indicating downregulated genes, red indicating upregulated genes, and black representing genes that showed no significant difference in expression. Heatmap of top 50 genes differentially expressed in control and CBL groups, blue represents lowly expressed genes and red represents highly expressed genes (Z-score); fold change (logFC), average expression (AveExpr) and gene name are shown (**C**).

To explore the impact of CBL as a biostimulant, we performed RNA-seq on tomato plants cultivated under control conditions, with foliar treatment using CBL, and without any treatment. In terms of the RNA-seq data, we assessed the variability among the replicates and across the treatments using a PCA plot (Figure 4A). This analysis revealed a clear clustering of replicates for the control group, while data points for the CBL-treated plants were scattered across the plot.

Moreover, the DEGs CBL vs. control were identified and it was predicted that the genes are involved in the response of plant to biostimulant. The volcano plot shows the distribution of DEGs at the threshold level for selection. Among 1300 DEGs, 907 and 393 were up- and downregulated in CBL vs. control, respectively (Figure 4B).

In addition, a heatmap from the 50 most expressed genes is shown in Figure 4C. The aggregated data heatmap analysis summarizes the responses of tomato plants to CBL application. This analysis identified two main clusters, corresponding to control and CBL-treated plants. In addition to the relative gene expression in each group, the fold change (logFC) of CBL vs. Control is shown, highlighting the average expression (AveExpr), which allows for the identification of the most highly expressed genes in the analyzed samples.

### 3.5. Functional Annotation and Enrichment of DEGs

**Figure 5:**
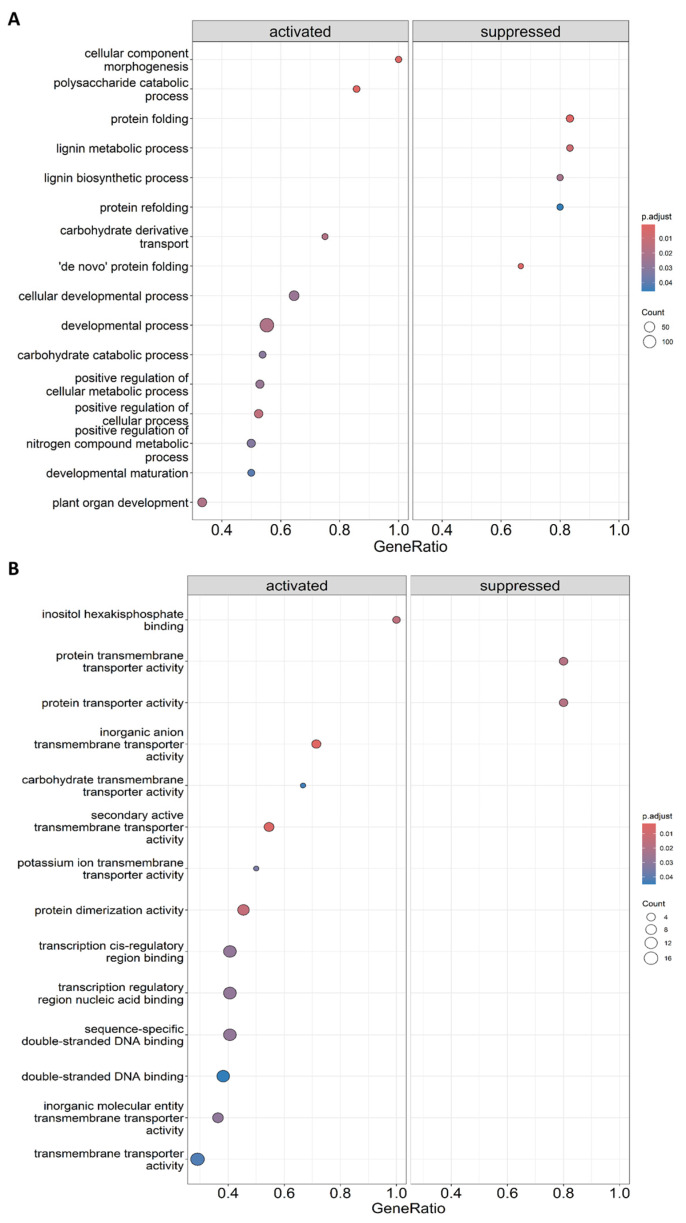
Gene ratio plot of the biological process (BP) terms (**A**) and molecular function (MF) (**B**) activated or repressed in CBL-treated plants in comparison with control plants. Items on the y axis are the names of KEGG terms, and the x axis represents the gene ratio. The depth of the color represents the adjusted *p*-value and the area of circles means gene counts.

In order to understand the functional implications of the gene expression alterations induced by the biostimulant CBL treatment, we conducted a functional annotation and enrichment analysis of the DEGs using Gene Ontology (GO) terms. In both the biological process (BP) and molecular function (MF) categories, we observed various terms enriched in CBL-treated plants (Table S1). Some of the terms related to development and plant growth were plotted in Figure 5 to provide a better understanding of the effects caused by CBL treatment. Among the activated BP terms, we identified “developmental process”, “carbohydrate catabolic process”, “positive regulation of cellular metabolic process”, and “plant organ development”. Additionally, we observed repressed terms such as “protein folding”, “protein refolding”, “de novo protein folding”, “lignin metabolic process”, and “lignin biosynthetic process” (Figure 5A, Table S2). On the other hand, in the MF category, we found several activated terms primarily related to transport activities, including “inorganic anion transmembrane transporter”, “secondary active transmembrane transporter”, “potassium ion transmembrane transporter,” and terms related to binding activities, such as “transcription cis-regulatory region binding”, “nucleic acid binding”, “inositol binding”, and “DNA binding” (Figure 5B, Table S2).

**Figure 6:**
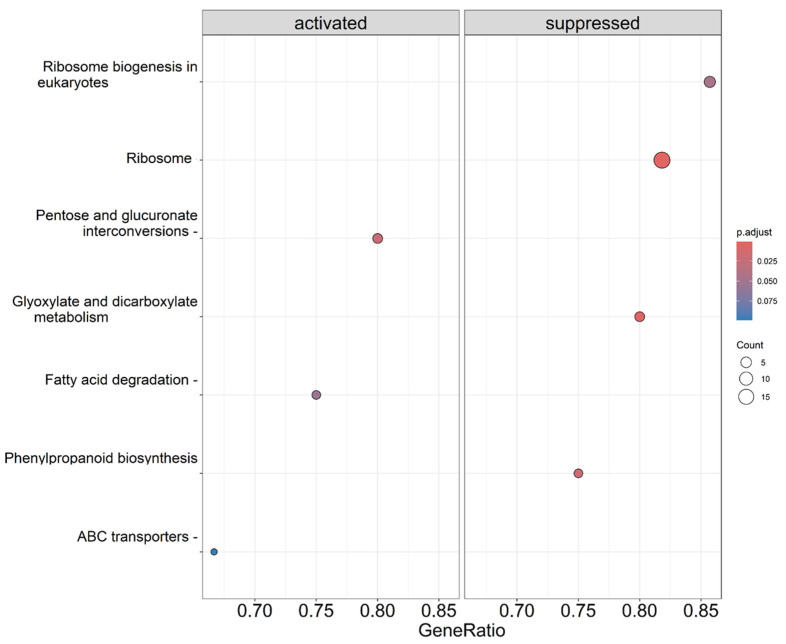
Gene ratio plot of the KEGG pathway activated or repressed in CBL-treated plants in comparison with control plants. Items on the y axis are the names of KEGG terms, and the x axis represents the gene ratio. The depth of the color represents the adjusted *p*-value and the area of circles means gene counts.

On the other hand, the same analysis was conducted for the KEGG pathways, and in this case, we observed several pathways that were positively enriched. These pathways include those related to “pentose and glucuronate interconversions”, “fatty acid degradation”, “phenylpropanoid biosynthesis”, and “ABC transporters”. In addition, pathways such as “ribosome biogenesis in eukaryotes”, “ribosome”, or “glycoxylate dicarboxylate metabolism” were repressed (Figure 6, Table S2).

## 4. Discussion

The utilization of biostimulants in modern agriculture has gained increasing importance in the context of evolving environmental conditions and the need for sustainable crop production. Our study delves into the application of flavonoids, specifically CropBioLife (CBL), as a potential biostimulant for tomato plants under optimal growth conditions. Research in this field of plant biostimulation is gaining interest in agricultural areas to study the biostimulant and signaling mechanisms that significantly enhance agricultural yields. Flavonoids, classified as secondary metabolites and biostimulants, have traditionally been associated with their pivotal role in promoting plant growth by increasing resistance to various biotic and abiotic stresses [22]. Their effects are often linked to protective mechanisms against factors such as UVB radiation [23], salt stress [3], and drought [24]. These protective effects arise from their ability to detoxify reactive oxygen species (ROS) generated during plant stress responses [25]. Additionally, some flavonoids have been found to act as natural defenses against certain pests and pathogens [26]. The stimulating effects of flavonoids on germination and growth observed in certain investigations can be attributed to allelopathy, a phenomenon associated with the synthesis of specific secondary metabolites [25]. Notably, several flavonoids, such as quercetin, isoquercitrin, and rutin, among others, have been shown to regulate plant growth [27], with the same effect being observed in the few studies in which flavonoids were externally applied [28].

In our previous results, we demonstrated the capacity of phenolic compounds present in CBL to promote plant growth by enhancing the exchange of CO_2_ and water within tomato leaves, consequently improving photosynthesis and transpiration. These alteration in cellular CO_2_ and water exchange were closely linked to the activity of aquaporins, which can trigger morphological alterations such as an increase in stomatal density on leaf surfaces [7]. Building upon these findings, in this current work, we determined that the higher growth of the shoot observed with external flavonoid application was associated with cell enlargement (Figure 1). These findings highlight the substantial impact of flavonoid biostimulant on the growth parameters of tomato plants, with a clear shift towards increased aboveground growth and larger individual cells. This phenomenon has not been documented previously, but it has been reported that increasing flavonoids is connected with the trafficking factor GFS9, which plays a crucial role in facilitating vacuolar development by mediating membrane fusion within vacuoles in *Arabidopsis*. This introduces the concept that plants utilize the GFS9-mediated membrane trafficking machinery not only for transporting proteins but also for delivering phytochemicals, such as flavonoids, to vacuoles, a process associated with cell enlargement [29]. This could be related to the increase in the flavonoid concentration and to the increase in the aquaporin functionality regulation observed [7].

Plant growth regulators, particularly auxins and cytokinins, are vital for various aspects of plant development and environmental stress responses. Auxins, such as IAA, and gibberellins, such as GA3, have been associated with not only promoting plant growth and seed germination but also enhancing the synthesis of flavonoids, as they upregulate the expression of mRNA specific to flavonoid production [30]. Cytokinins, another class of plant growth regulators, are known for their multifaceted roles, including promoting cell division, controlling meristem activity, facilitating organogenesis, regulating leaf senescence, directing vascular differentiation, and aiding nutrient acquisition [8]. Additionally, cytokinins have been demonstrated to play a significant role in enhancing plant resistance to abiotic stress, particularly increasing drought tolerance [31].

The generation and accumulation of zeatin (a major endogenous cytokinin in plants) can promote cell division to enhance plant self-repair after exogenous application [32]. In addition, CBL positively affected the zeatin levels in the tomato leaves as vital phytohormone in promoting stem elongation [33]. However, GA3 was not detected in our plants in either control or in CBL-treated plants. Moreover, there was a noteworthy reduction in IAA levels in the leaves of CBL-treated plants (Figure 2). The zeatin/IAA relationship was significant in our study. In previous research, it has been reported that variations in the zeatin/IAA relationship within meristematic cells can influence the choice between endoreduplication and cytokinesis [34]. The observed alteration in the zeatin/IAA relationship in CBL-treated tomato tissue points towards increased cell growth, which could underlie the growth-related effects of CBL. The increased expression and functionality of aquaporins previously reported in CBL-treated plants [7] may be intrinsically tied to the improved water uptake associated with cytokinesis, in line with previous research. Accordingly, Chapman and Estelle [35] observed that cytokinin regulated meristem size in the root by antagonizing auxin signaling in the transition zone. The region where cells leave the meristem to elongate and differentiate should be highly influenced by the water status of the plant. Collectively, the physiological and biochemical findings, together with the multivariate analysis (Figure 3), distinctly demonstrate a positive correlation between CBL and various factors, including phenolic compounds, photosynthesis, biomass, and zeatin.

Valente et al. [36] also observed that auxin was able to induce DNA expression, which finally resulted in endoreduplication in the absence of cytokinin, but cytokinin was required for mitosis. Our investigation revealed a higher number of upregulated genes compared to those that were repressed by CBL treatment (Figure 4). The enhanced gene expression associated with biological processes such as “developmental process”, “cellular developmental process”, and “carbohydrate catabolic process” aligns with the findings regarding increased chlorophyll concentration, enhanced fluorescence of photosystem II, and elevated internal CO_2_ concentration observed in our previous research [7]. Specifically, our RNA-seq analysis revealed the overexpression of the tomato *JAGGED* homologue (*LYRATE*) (gene id: 843177) in CBL-treated plants. *LYRATE*, known as a significant positive regulator of cell division in lateral organs [37], falls within the “cellular developmental process” category. In fact, mutations and the *LYRATE* locus in tomato affected the proper development of floral organs and fruit formation [38]. The observed overexpression of this gene could partially elucidate the increased growth observed in CBL-treated plants. Moreover, LYRATE’s role in modulating auxin response and distribution is noteworthy, as these processes were also affected in our plants treated with the CBL biostimulant. Furthermore, the differentially expressed genes related to molecular functions included several upregulated genes linked to transport activities, including inorganic anion transmembrane transporter, secondary active transmembrane transporter, and potassium ion transmembrane transporter. Within these upregulated categories, we find a gene of the MATE family, specifically the *DTX41* gene (gene id: 825072). The proteins encoded by the MATE family genes have been shown to regulate overall plant development through the transport of phytohormones, ion homeostasis, tip growth processes [39] and even flavonoids, since they act as transporters from the site of flavonoid biosynthesis towards the vacuole and other subcellular compartments [40]. Related to ion transport, a gene that codifies a cation–chloride cotransporter (CCC) (gene id: 839924) is also upregulated, which operates as an Na^+^:K^+^:Cl^−^ cotransporter and has been insolvent in developmental processes [41]. Altogether, this information aligns with the well-established interplay between mineral uptake, plant water status, and growth [42]. While both ions and water are absorbed through specialized membrane transporters, including channels, antiporters, and ion transporters, as well as aquaporins for water, it was demonstrated that the regulation of these transporters is coordinated to maintain cellular homeostasis.

Thus, the observed increase in growth in CBL-treated plants is intricately tied to elevated mineral transport and enhanced water uptake. In these processes, the role of MATE family genes is pivotal. Recently, these genes have been identified as voltage-dependent chloride anion channels, facilitating chloride entry into the vacuole. They play a crucial role in regulating turgor pressure and cell expansion and act as key regulators in significant processes such as stomatal movements in guard cells [43,44]. This finding is consistent with our previous work, where we demonstrated the impact of elevated internal CO_2_ concentration in plants treated with CBL. Moreover, in relation to water uptake, it is essential that MATE transporters and aquaporins function in a coordinated manner. Stomatal opening and closing are finely regulated, involving the interplay of aquaporins, MATE transporters, and other agents [44].

In addition to the transcriptomic changes, the KEGG analysis provide several pathways positively enriched as “fatty acid degradation”, “phenylpropanoid biosynthesis”, and “ABC transporters” (Figure 6). Cell division and enlargement represent the predominant processes during the initial phase of growth, concomitant with the production and buildup of organic acids, methoxypyrazines, and phenolic compounds [45]. The regulation of this phase in tomato has been related to auxins and cytokinins, as has also been shown in our work. According to the “fatty acid degradation pathways”, previous research on the impact of synthetic cytokinins on grapes observed more highly downregulated genes associated with fatty acid biosynthesis [46]. In relation to phenylpropanoids, these comprise an extensive category of plant secondary metabolites originating from aromatic amino acids, primarily phenylalanine in the majority of plants or tyrosine in certain monocots. The primary branches of the phenylpropanoid pathway encompass lignans and lignins, stilbenes, coumarins, isoflavonoids, flavonoids, and proanthocyanidins [47]. Therefore, the concurrent increase in gene expression related to phenylpropanoid biosynthesis is closely linked to the observed elevation in flavonoid concentration in tomato leaves, as detailed in our earlier work [7].

## 5. Conclusions

Our findings underscore the significance of external flavonoid application, specifically the biostimulant CropBioLife (CBL), in the context of optimal tomato plant growth. We observed a complex interplay between flavonoid application and induced cell enlargement. Effective flavonoid (CBL) application revealed a regulatory mechanism related to hormones, as we noted an increase in zeatin concentration followed by a decrease in IAA concentration. Furthermore, we identified a positive correlation between CBL and phenolic compounds, photosynthesis, biomass, and zeatin. Our RNA-seq analysis also showed that CBL-treated plants should increase mineral uptake and transport, as suggested by the increase in the expression of genes related to the transport activity of inorganic ions and membrane transporters. Additionally, genes associated with fatty acid degradation pathways, phenylpropanoid biosynthesis, and ABC transporters exhibited regulatory mechanisms governing internal flavonoid biosynthesis, transport, and tissue concentration, ultimately resulting in higher flavonoid levels in tomato leaves. Therefore, our results revealed that the upregulation of genes related to flavonoid synthesis and transport, along with those associated with mineral nutrient and water uptake, are key mechanisms shaping the response of tomato plants to external flavonoid application under optimal growth conditions. However, the intricate relationship between flavonoids and the promotion of cell growth in plants from a biostimulant perspective need continued investigation.

## Data Availability

Data are contained within the article and Supplementary Materials.

## Supplementary Materials

The following supporting information can be downloaded at https://www.mdpi.com/article/10.3390/genes14122208/s1. Table S1: List of genes differentially expressed. Table S2: Biological process (BP), molecular function (MF) categories, and KEGG pathways enriched in CBL-treated plants.
